# From oral erosions to lymphoma: a case of paraneoplastic pemphigus with occult lymphoma

**DOI:** 10.1093/omcr/omaf124

**Published:** 2025-08-11

**Authors:** Kumari Monalisa, Tanvi Dev, Vaishnavi Modi, Akarshi Gupta, Varuna Mallya, Rashmi Dixit

**Affiliations:** Specialist Medical Officer (Department of Dermatology), UPHC Uttarwari Pokhra, Bettiah, West Champaran, Bihar, 845438, India; Department of Dermatology, Maulana Azad Medical College & Lok Nayak Hospital, Bahadur Shah Zafar Marg, New Delhi 110002, India; Department of Dermatology, Maulana Azad Medical College & Lok Nayak Hospital, Bahadur Shah Zafar Marg, New Delhi 110002, India; Department of Radiology, Maulana Azad Medical College & Lok Nayak Hospital, Bahadur Shah Zafar Marg, New Delhi 110002, India; Department of Pathology, Maulana Azad Medical College & Lok Nayak Hospital, Bahadur Shah Zafar Marg, New Delhi 110002, India; Department of Radiology, Maulana Azad Medical College & Lok Nayak Hospital, Bahadur Shah Zafar Marg, New Delhi 110002, India

**Keywords:** pemphigus, paraneoplastic, lymphoma, oral erosions

## Abstract

A 67-year-old female with type-2 diabetes mellitus presented with recalcitrant painful oral erosions and violaceous chest lesions, that responded minimally to oral corticosteroids. Due to history of decreased appetite, weight loss and poor therapeutic response, further evaluation was performed, revealing elevated C-Reactive Peptide, Desmoglein-3 antibody, Carbohydrate Antigen 19–9, and intraperitoneal lobulated homogeneously enhancing nodal mass, the latter indicating lymphoma. Histopathology from oral mucosa was suggestive of paraneoplastic pemphigus. The patient was referred to oncology department for further management. This case underscores the importance of thorough evaluation in elderly patients with recalcitrant mucocutaneous lesions with atypical histopathology.

## Introduction

Paraneoplastic pemphigus (PNP) is a rare autoimmune blistering disorder associated with underlying neoplasm. The clinical presentation can mimic other mucocutaneous diseases, posing diagnostic challenges. We describe a case of a 67-year-old female who presented with refractory, painful oral erosions and subsequently developed dusky erythematous cutaneous lesions. The patient's condition persisted despite treatment, leading to further investigations that revealed an underlying lymphoma. This case underscores the importance of suspecting the possibility of an underlying malignancy in cases with refractory mucosal erosions and atypical cutaneous lesions, especially in the elderly population.

## Case report

**Figure 1:**
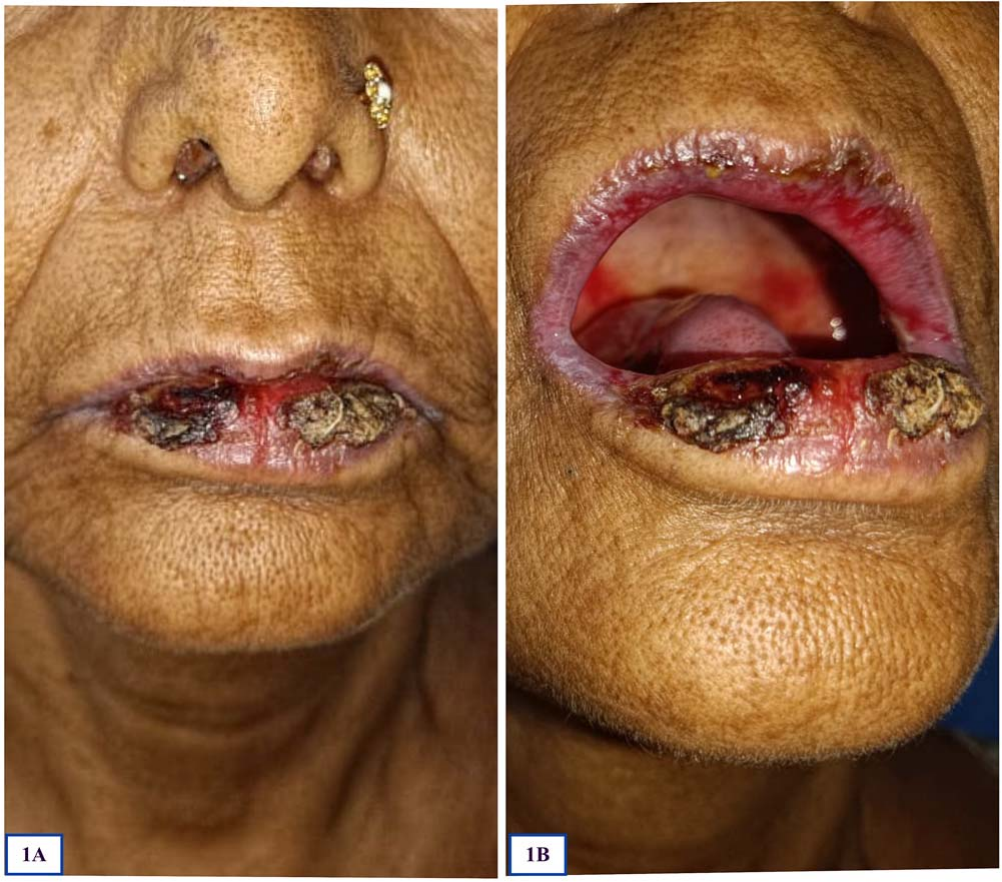
(A) and (B) showing hemorrhagic, crusted erosions involving lips, buccal mucosa and hard palate.

A 67-year-old-female, who was a known case of type-2 diabetes mellitus, presented with multiple painful erosions in the oral cavity for 1 month, along with few dusky erythematous lesions over chest for 2 weeks (Fig. 1A and B). The erosions in the oral cavity were persistent and involved lips, gingiva, buccal mucosa and pharynx, with some lesions exhibiting bleeding upon touch. Painful deglutition was noted. Subsequently, few dusky erythematous lesions appeared on her chest, some displaying overlying erosions. There was no past history of similar complaints. Bowel and bladder history was unremarkable and her sleep was adequate. There was history of decreased apetite, recent undocumented weight loss and low-grade fever for 3 months. There was no history of intake of any medication prior to the onset of the disease.

**Figure 2:**
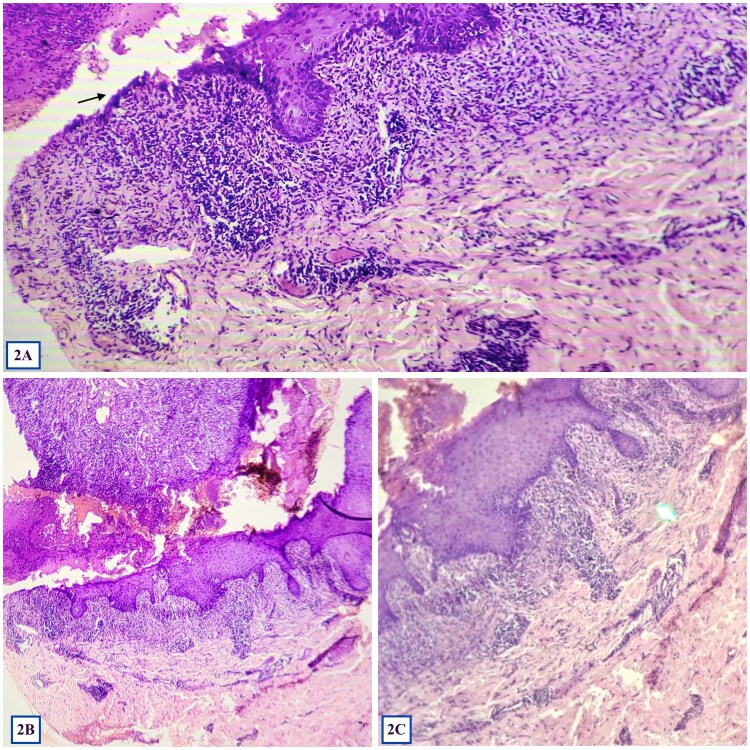
(A), (B), (C) showing biopsy from oral mucosa revealing complete epidermal necrosis with lymphocytic exocytosis along with suprabasal split, ‘row of tombstone’ appearance (marked with black arrow) at one end of the section, and confluent dense lymphocytic lichenoid infiltrate in the superficial dermis at 100× and 400× magnification. (Hematoxylin and eosin).

The patient had thin built but there was no pallor, icterus or lymphadenopathy. Cutaneous examination revealed multiple discrete to coalescent tender erosions over lips, bilateral buccal mucosa, and pharynx, with some lesions on the lips displaying hemorrhagic crust. Additionally, multiple erythematous to violaceous discrete papules were noted over chest and abdomen, with some showing overlying erosions. Scalp, nails, palms and soles were unremarkable. Clinically, the differential diagnosis of pemphigus vulgaris, Steven Johnson syndrome (SJS), and erosive lichen planus were considered.

Histopathology from oral mucosa revealed complete epidermal necrosis with lymphocytic exocytosis along with one end of the section showing suprabasal split with a ‘row of tombstone’ appearance, and confluent dense lymphocytic lichenoid infiltrate in the superficial dermis (Fig. 2A–C). Due to financial limitations and poor resource availability, direct immunofluorescence could not be done. Blood investigations showed unremarkable hemogram and liver and kidney function tests and elevated C-Reactive Peptide (CRP: 228 mg/dl; normal range: 0.3-1 mg/dl), Desmoglein-3 antibody (691.2 U/ml; normal range: < 20 U/ml) and Carbohydrate Antigen 19–9 (CA 19–9:107 U/ml; normal range: < 37 U/ml). Peripheral smear, Papanicolaou smear, and occult blood stool test were unremarkable. Abdominal ultrasonogram revealed fatty liver changes only. With the diagnosis of pemphigus vulgaris based on histopathology and elevated desmoglein-3 antibody levels, she was treated with oral prednisolone (1 mg/kg), along with triamcinolone oral mouth paint and chlorhexidine mouth rinses, however there was a minimal improvement noted after 4 weeks of treatment.

**Figure 3:**
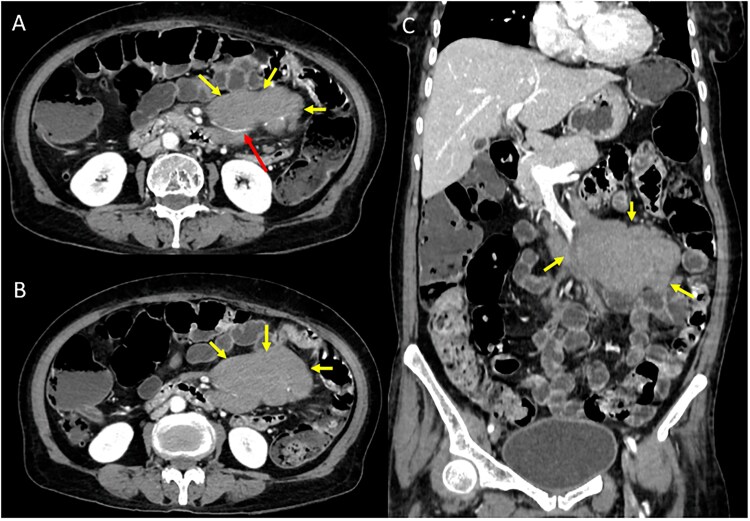
Contrast-enhanced Computed Tomography (CECT) axial (A,B) and coronal (C) images reveal intraperitoneal lobulated homogeneously enhancing nodal mass in the mesentery encasing branches of superior mesenteric artery without luminal attenuation giving ‘Sandwich sign’ suggestive of lymphoma.

Due to the patient's advanced age, refractory muco-cutaneous lesions, elevated CA 19–9 and unusual lichenoid infiltrate on the biopsy, a possibility of PNP was considered. Contrast-enhanced Computed Tomography (CECT) scan of chest and abdomen revealed intraperitoneal lobulated homogeneously enhancing nodal mass in the mesentery encasing branches of superior mesenteric artery without luminal attenuation giving ‘Sandwich sign’ suggestive of lymphoma (Fig. 3A–C). Oral prednisolone was continued and the patient was transferred to oncology department. Histopathological examination of the intraperitoneal lymph node could not be performed as the patient succumbed to her illness before any invasive diagnostic intervention could be undertaken.

## Discussion

PNP is a rare, life-threatening autoimmune blistering disorder, often associated with an underlying neoplasm, particularly lymphoproliferative malignancies such as non-Hodgkin’s lymphoma, chronic lymphocytic leukemia and Castleman’s disease. The diagnosis of PNP is done by various diagnostic criteria. The most accepted being the ‘Camisa & Helm’ criteria (Table 1) [1]. PNP can affect multiple organ systems, leading to respiratory and gastrointestinal complications.

**Table 1:** ‘Camisa & Helm’s criteria’ for the diagnosis of paraneoplastic pemphigus

Major criteria	Minor criteria
Polymorphic mucocutaneous eruption. Concomitant internal neoplasm. Serum antibodies with a specific immunoprecipitation reaction.	Acantholysis observed histopathologically. DIF displaying intercellular and basement membrane staining. IIF staining positive on rat bladder epithelium.
The diagnosis of paraneoplastic pemphigus requires fulfilling either of the following criteria: Three major criteria Two major and two minor criteria	

This case highlights the diagnostic challenges of PNP due to its overlapping clinical and histopathological features with other autoimmune blistering disorders, like pemphigus vulgaris, SJS, and erosive lichen planus [2, 3].

The present case is of an elderly female who presented with painful refractory oral erosions and dusky erythematous lesions on the chest which were acute in onset. Recalcitrant oral erosions, as observed in the present case, unresponsive to treatment, are a hallmark feature of PNP. The associated cutaneous findings, including lichenoid and erosive lesions, further align with the polymorphic presentation of this condition. Notably, several cases of PNP have been described in the literature where patients present with an atypical blistering condition, constituted by painful blisters and erosions of the oral mucosa, occaisionally being the sole manifestation of the disease [4, 5]. This underscores the need to identify cases of PNP in the absence of exuberant peri-oral and peri-ungual lesions.

Despite initial treatment with systemic corticosteroids, the patient exhibited only partial improvement. This therapeutic resistance is characteristic of PNP, distinguishing it from pemphigus vulgaris, which generally shows a better response to steroids [6].

The histopathological findings of dense lichenoid infiltrate in combination with epidermal necrosis, supra-basal split and ‘row-of-tombstone’ appearance pointed towards the possibilty of PNP. Elevated CRP level suggested ongoing systemic inflammation, while the raised desmoglein-3 antibody levels indicated autoimmune blistering, favoring a pemphigus variant. CA 19–9 is a screening marker for intra-abdominal solid organ malignancies, however it can also be elevated in lymphoma, which was confirmed on abdominal CECT [7].

Anti-plakin antibodies, including those against envoplakin and periplakin, are recognized as specific immunological markers for PNP and are incorporated into the established diagnostic criteria. However, their appearance is often delayed, and they may be absent in the early stages of the disease, potentially leading to diagnostic challenges [8, 9].

Recent evidence suggests that autoantibodies against desmoglein typically emerge earlier in the disease course and may play a pivotal role in initiating the pathogenic cascade. The disruption of keratinocyte adhesion by desmoglein 3 antibodies is hypothesized to expose intracellular plakin antigens, thereby triggering a secondary immune response and subsequent production of anti-plakin antibodies [10].

In the present case, the patient unfortunately succumbed before this immunological sequence could fully evolve, underscoring the temporal dynamics of autoantibody development in PNP and the importance of early recognition and intervention.

This case emphasizes the importance of a thorough systemic evaluation in non-responding mucocutaneous lesions, particularly in elderly patients, to rule out paraneoplastic syndromes. High degree of suspicion and a multidisciplinary collaboration is crucial for the prompt diagnosis and management of this condition.
